# Insights on the Role of Antimicrobial Cuffed Endotracheal Tubes in Preventing Transtracheal Transmission of VAP Pathogens from an *In Vitro* Model of Microaspiration and Microbial Proliferation

**DOI:** 10.1155/2014/120468

**Published:** 2014-04-10

**Authors:** Joel Rosenblatt, Ruth Reitzel, Ying Jiang, Ray Hachem, Issam Raad

**Affiliations:** Department of Infectious Diseases, Infection Control and Employee Health, Unit 1460, The University of Texas MD Anderson Cancer Center, 1515 Holcombe Boulevard, Houston, TX 77030, USA

## Abstract

We developed an *in vitro* model to evaluate the effect of different cuffed endotracheal tubes (ETTs) on transtracheal transmission of ventilator-associated pneumonia (VAP) pathogens along external surfaces of ETTs. The model independently assessed the relative contributions of microbial proliferation to the distal tip and microaspiration of contaminated secretions past the cuff by testing in three modes: microaspiration only, microbial proliferation only, and simultaneous microaspiration and microbial proliferation. We evaluated transmission of methicillin resistant *Staphylococcus aureus* (MRSA) or *Pseudomonas aeruginosa* (PA) in the presence of a standard ETT; a soft, tapered cuff ETT with subglottic suctioning; and a novel antimicrobial gendine (combination of gentian violet and chlorhexidine) ETT in the model. In the microaspiration only mode, when leakage past the cuff occurred quickly, no ETT prevented transmission. When microaspiration was delayed, the gendine ETT was able to completely disinfect the fluid above the cuff and thereby prevent transmission of pathogens. In microbial proliferation only mode, the gendine ETT was the sole ETT that prevented transmission. With both mechanisms simultaneously available, transmission was dependent on how long microaspiration was delayed. Potent antimicrobial ETTs, such as a gendine ETT, can make unique contributions to prevent VAP when microaspiration is gradual.

## 1. Introduction

Ventilator-associated pneumonia (VAP) is a serious health care issue that adds significantly to hospital costs [1, 2]. In order for VAP to occur, there needs to be significant microbial contamination of the normally sterile lung tissues [3, 4]. Endotracheal tubes (ETTs) used in ventilator circuits provide a conduit for pathogenic microbes to pass from the heavily colonized oropharynx to the normally sterile lungs [5]. There are two primary modes of transit associated with ETTs. The secretions can come from refluxed gastric fluids or from saliva that is produced in the oropharynx due to the presence of the foreign ETT. The cuff creates a boundary at the cuff-tracheal junction where secretions can pool. If channels or folds are present in the cuff at the tracheal junction the pooled contaminated secretions can flow under force of gravity or by negative pressures created during inspiration through the channels into the bronchioles and lungs (microaspiration). This convection can be reduced by use of subglottic suctioning which reduces the volume of the pooled secretions that can leak through [6]. It can also be reduced by more compliant cuff materials or cuff designs that ensure complete circumferential contact and minimize channel formation due to cuff wrinkling or folding [7]. A second transit pathway is microbial proliferation along the ETT around the cuff to the distal tip of the ETT [8]. Condensate or mucus can form on the colonized ETT and by dripping back into the bronchioles or deep lung carrying microbes to these normally sterile areas. The Centers for Disease Control and Prevention have recommended several patient- and procedure-based practices that can be implemented to reduce the incidence of VAP, including regular oral disinfection, inclining the patient to reduce reflux, daily assessment of the need for continued intubation, and early tracheostomies for long-term intubations [9]. Although the National Healthcare Safety Network has reported significant reductions in the incidence of VAP, mortality rates associated with this infection are still higher than those associated with any other hospital-acquired infection [10].

In addition to procedural bundles, several other endotracheal tube based innovations have targeted reductions in the incidence of VAP, including modified cuff designs and materials, subglottic suctioning, and antimicrobial coatings. A recent review concluded that the clinical evidence to support the routine use of these innovations was insufficient [11]. Two reasons for the conclusions presented in the review were limited sample sizes in the clinical trials and equivocal definitions used for VAP attribution. Other reviews arrived at similar conclusions [12, 13]. Two recent meta-analyses, however, showed that subglottic suctioning was associated with reduced risk for VAP and a shorter duration of mechanical ventilation [14, 15]. Because of the cost and difficulty involved in obtaining informed consent from intubated patients in human clinical trials,* in vitro* and* ex vivo* models were developed to elucidate and demonstrate the mechanisms by which enhanced ETT designs can reduce VAP. Some of the tested ETT innovations have included tapered cuff designs, thin-walled elastomeric cuffs, and various subglottic suctioning systems. Several models have focused on the leakage of pooled, microbially contaminated secretions from the oropharynx around the cuff that were aspirated into the lungs [16–22]. Other models have considered biofilm colonization on tubes and were applied to tubes with antimicrobial coatings in order to test their ability to inhibit microbial proliferation to the distal tip of the ETTs [23, 24].

We developed a simplified model to assess the impact of different ETTs on both microaspiration of contaminated pooled secretions around the cuff and microbial proliferation along the external surface of the ETT as valid dual mechanistic pathways for microbes to access the lungs and cause VAP. Microbial proliferation can occur along both the external and lumenal surfaces of the ETT. Microbes can colonize the lumenal ETT surface if the ventilator circuit is broken and microbial contact is allowed. Lumenal colonization can be minimized and prevented by rigorous care and handling precautions. In the model, microbial proliferation along the lumenal surfaces of the ETT was prevented by precluding microbial contact with the lumen as long as the lungs remained sterile. Microbes colonize the external ETT surface through contact of the ETT with contaminated oropharyngeal secretions (this contact is always present). Specifically we assessed the ability of a standard ETT, a tapered cuff subglottic suctioning ETT, and a novel antimicrobial gendine (combination of gentian violet and chlorhexidine) standard ETT to inhibit transtracheal transmission of pathogens. Clinical and* in vitro* studies of a silver-coated tube have shown that colonization can be reduced and rates of VAP decreased but not eliminated [25]. We, therefore, utilized a gendine ETT, since it has been shown to be more potent than silver in preventing adherence of multidrug-resistant bacteria on ETTs [26]. By operating the model in modes which isolated each pathway, we assessed how effective the different ETTs were in inhibiting microbial transmission through the trachea by either or both mechanisms.

## 2. Materials and Methods

### 2.1. Model Description

#### 2.1.1. Physical Description

**Figure 1:**
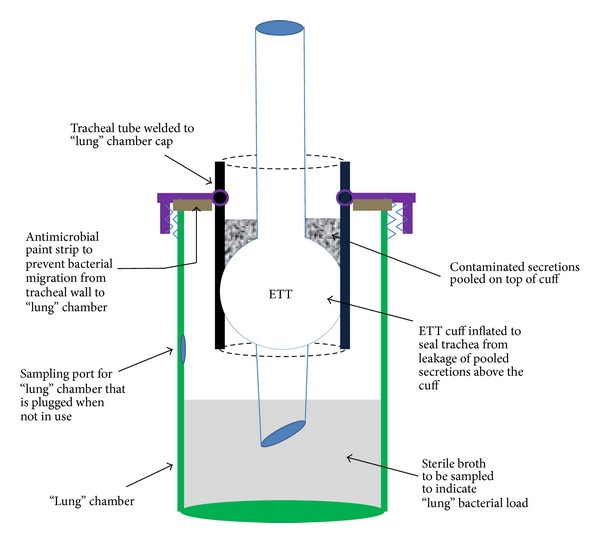
Diagram of* in vitro* model for simultaneous secretion-aspiration and biofilm-colonization as pathogenic routes to contamination of “lungs.”

An ETT-trachea model was developed as depicted in Figure 1. The trachea consisted of a short polypropylene tube of 2.5 cm inner diameter and 10 cm length. A larger flat-bottomed container (simulating the lungs) consisted of a 500 mL polystyrene vessel, 13 cm high with a 4 cm diameter screw cap. A 2.7 cm hole was cut in the cap and the polypropylene tube was welded inside the cap such that 7 cm protruded below the cap and 3 cm above. There was a gap of 5 cm between the bottom of the vessel and the base of the tube when the welded cap-tube assembly was screwed together. A 5 mm diameter sampling port was cut into the wall of the vessel about 6 cm above the base. This could be plugged with a rubber or plastic stopper. An ETT could be suspended in the tube and held in place with a clamp such that the tip of the tube extended below the base of the tube and 4 cm above the base of the vessel. In this position, a fluid volume of 300 mL in the vessel would completely submerge the tip of the ETT and there would be no tip-fluid contact for a fluid volume of 150 mL. Antimicrobial conductive silver paint was applied to the underside of the cap to prevent microorganisms from proliferating down the sides of the vessel and contaminating the lungs (hence, the only proliferative pathway available was along the external surfaces of the ETT).

#### 2.1.2. Description of Simulated Anatomy

The polypropylene tube simulated the trachea and the larger flat-bottomed vessel simulated the lungs. When the cuff of an ETT suspended within the tube was inflated to 30 cm H_2_O (normal pressure) it circumferentially contacted the trachea. If overinflated (100 cm H_2_O), the cuff formed a hydraulic seal with the trachea. With the cuff inflated, liquid could be pipetted above the cuff where it pooled, simulating pooled oropharyngeal secretions. Microorganisms could be added to these “secretions” creating simulated pooled contaminated secretions. Prior to initiating an experiment, the entire model assembly was ethylene oxide sterilized. Upon adding the “pooled contaminated secretions” above the cuff, microbes could access the sterile “lung” chamber below the cuff either by gravitationally driven leakage between the cuff and trachea (simulating microaspiration) or by microbial proliferation along the ETT around the cuff. Sterile liquid (broth) added to the “lung” vessel simulated bronchoalveolar fluid. This could be sampled through the sampling port for analysis at different time points. The lumen of the ETT was sterile until the lung chamber became contaminated as no microbial contact with the lumen occurred as long as the lung chamber was sterile.

#### 2.1.3. ETTs Tested and Cuff Inflation Pressure Monitoring

Three ETTs were used in the experiments with the model: (1) a standard polyvinylchloride ETT was used as a control (ID of 7.5 mm, OD of 10.2 mm, and inflated cuff OD of 24 mm, Sheridan, Teleflex Medical, Research Triangle Park, NC); (2) an advanced design ETT with soft, tapered polyurethane cuff and subglottic suctioning designed to minimize microaspiration (ID of 7.5 mm, OD of 11.2 mm, and inflated cuff OD of 26 mm, Sealguard, Covidien Inc., Mansfield, MA); and (3) a standard polyvinylchloride ETT treated with gendine as previously described [27] on both the cuff and shaft. To initiate an experiment, an ETT was inserted into the mock trachea and inflated to a desired pressure. Fluid inoculated with bacteria could then be pipetted above the cuff to simulate pooling of contaminated secretions and transmission through the trachea monitored. In certain dye transmission experiments, the pooled secretions above the cuff were suctioned down to the level of the suctioning port when the subglottic suctioning capable ETT was used. During the course of an experiment, the cuff pressure was measured and adjusted several times daily with a cuff pressure gauge (Cufflator, JT Posey, Arcadia, CA).

### 2.2. Microbiological Methods

#### 2.2.1. Organisms

Clinical isolates of methicillin resistant* Staphylococcus aureus* (MRSA) and multidrug-resistant* Pseudomonas aeruginosa* (PA) were tested independently to determine their ability to colonize around the ETT cuff and down into the larger container simulating the lungs. These organisms were selected as model VAP pathogens in this study because they are responsible for the greatest incidences of VAP [4].

#### 2.2.2. Contaminated Secretions

Isolates were freshly grown on trypticase soy agar + 5% sheep blood, inoculated into Mueller-Hinton II broth (Fischer Scientific, Pittsburg, PA) and diluted to a concentration of 5.5 × 10^5^ colony-forming units per milliliter for performing contaminated secretion bacterial challenges. This concentration was chosen as a worst case scenario and was greater than the 1 × 10^4^ bronchoalveolar lavage concentration used to define VAP in previous studies [17]. The viscosity of the pooled contaminated challenge fluid also represented a worst case scenario as it was water-like and lower than proteinaceous sputum. After the cuff was inflated to the desired pressure, 20 mL of bacterial inoculum was pipetted into the mock trachea on the top (proximal) side of the cuff to perform a contaminated secretion challenge. This volume was chosen to simulate a worst case large quantity of secretions pooling above the cuff to create significant gravitational driving forces for leakage as negative pressures were not applied. The 20 mL volume also provided a sufficient quantity for daily sampling for quantitative culture (see below). The top of the trachea and upper opening of the ETT were sealed with occlusive film to prevent contamination and evaporation. Testing was performed at 37°C.

#### 2.2.3. Sampling and Quantitation of Secretions above the Cuff or from the Lungs

Fluid from above the cuff as well as from the lower (lung) reservoir was sampled for testing using a 2 mL transfer pipette after 24, 48, 72, and 144 hours of incubation for bacterial growth. Leakage was assessed daily throughout the duration of an experiment by visually checking the volume of broth above the cuff. The pressure of the cuff was checked and adjusted several times daily. Bacterial growth was quantified at each time point by withdrawing 1 mL from either compartment, serially diluting it, and quantitatively culturing the dilutions onto trypticase soy agar + 5% sheep blood for growth.

### 2.3. Model Operating Modes for Transtracheal Transmission of Pathogens

#### 2.3.1. Microaspiration Only

*(A)   Dye Experiments.* After gas sterilization of the model assembly, sterile Mueller-Hinton II broth was pipetted into the lower reservoir. An ETT was inserted and inflated to the desired pressure. 20 mL of broth to which colored dye had been added was pipetted above the inflated cuff in the model. Cuff inflation pressure was checked and adjusted several times daily. The lower reservoir was visually inspected at the same times to see if the color of the broth had changed due to dye leaking past the cuff-tracheal junction.

**Figure 2:**
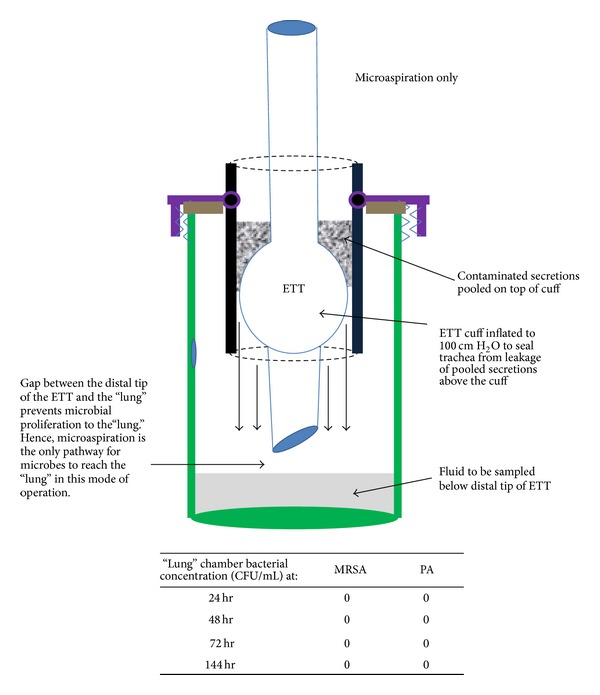
Diagram of the model run in microaspiration only mode where these is a gap between the distal tip of the ETT and fluid in the lung chamber. A verification experiment with quantitative culture was run showing that microbial proliferation is precluded in this mode. In the verification experiment, a standard ETT was used and the cuff was inflated to 100 cm water to preclude microaspiration. The gap between the distal tip of the ETT and the initially sterile fluid in the lung chamber broke the continuous surface pathway required for MRSA (methicillin resistant* Staphylococcus aureus*)and PA (*Pseudomonas aeruginosa*) to proliferate along the ETT to the lung chamber; hence, it remained sterile for 144 hours.

*(B)   Lung Fluid below the Level of the Distal Tip of the ETT.* The lower reservoir (lungs) was filled with only 150 mL of sterile broth such that the distal tip of the ETT did not contact the sterile broth (see Figure 2). This air gap prevented microbes from reaching the broth in that chamber by proliferation along the shaft of the ETT distal to the cuff. Therefore, leakage past the cuff-tracheal junction was the only pathway for bacteria to reach the 150 mL of originally sterile broth in the lower (lung) chamber.

#### 2.3.2. Microbial Proliferation Only

*(A)   Dye Experiments.* Experiments using colored dye added to the broth simulating pooled contaminated secretions above the cuff were conducted to determine the cuff inflation pressure required to ensure no microaspiration (leakage) occurred over the course of a 144-hour experiment. Cuff inflation pressures of 100 cm H_2_O were required to fulfill this condition. With microaspiration precluded due to high cuff inflation pressures microbial proliferation along the external surface of the ETT was the only pathway for bacteria to reach the lower (lung) reservoir.

**Figure 3:**
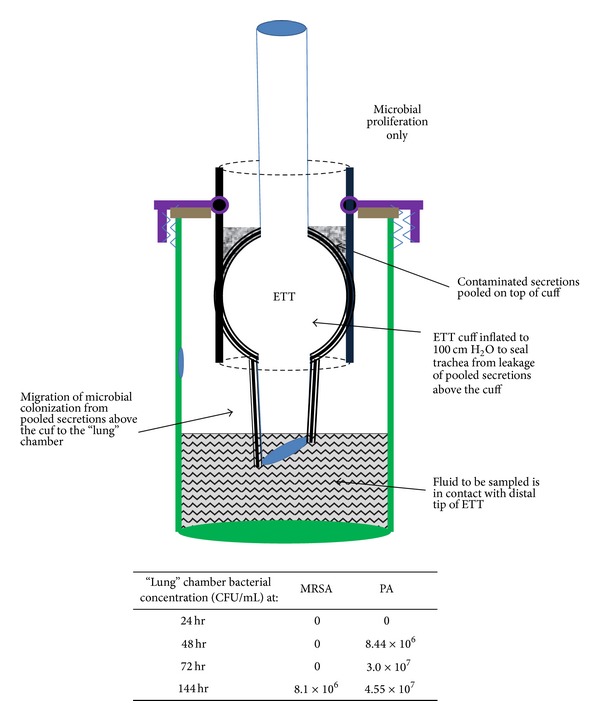
Diagram of the model run in microbial proliferation only mode and quantitative culture showing time required for the lung chamber to become contaminated in this mode. In microbial proliferation only mode microaspiration is precluded by overinflating the cuff to 100 cm H_2_O. Dye experiments verified that no leakage occurred over 144 hours at this inflation pressure. Quantitative culture results show times required to contaminate the “lung” chamber using a standard ETT with MRSA (methicillin resistant* Staphylococcus aureus*) and PA (*Pseudomonas aeruginosa*) inocula introduced above the cuff.

*(B)   Sampling above the Cuff and from the Lungs.* Experiments were performed at the high cuff inflation pressures (100 cm H_2_O) to hydraulically seal the trachea. Broth inoculated with either MRSA or PA was pipetted on top of the cuff. The lower (lung) reservoir was filled with 300 mL initially sterile broth such that the distal shaft of the ETT contacted the fluid in the lower reservoir (see Figure 3). Aliquots of 1 mL of fluid from both above the cuff and from the lower reservoir were sampled daily and quantitatively cultured. Maintenance of sterility in the lower reservoir indicated that microbial proliferation along the shaft of the ETT had not yet reached the distal tip. The time point at which a positive culture occurred in the lower reservoir was taken as the time required complete colonization of the distal shaft of the ETT by microbial proliferation.

#### 2.3.3. Simultaneous Microaspiration and Microbial Proliferation

Experiments run at 30 cm H_2_O cuff inflation pressures enabled both microaspiration and microbial proliferation pathways to operate simultaneously for bacteria to reach the lower (lung) reservoir.

### 2.4. Statistical Methods

Statistical analyses were performed by using SAS version 9.3 (SAS Institute Inc., Cary, NC) to compare bacterial concentrations above and below the cuff for the various ETTs. For each organism, comparisons were performed by using two-way nonparametric ANOVA (using ANOVA in conjunction with rank transformation). Concentrations at a single time point were compared by using Wilcoxon rank sum test. All tests were two-sided at a significance level of 0.05 to compare bacterial concentrations above and below the cuff for the various ETTs. All comparative experiments were performed in quadruplicate.

## 3. Results

### 3.1. Dye Leakage Results (Immediate)

For normal cuff inflation pressures (20–30 cm H_2_O), leakage of dyed broth to the lower reservoir was seen within 48 hours. A pressure of 100 cm H_2_O was determined (from visualization of dye transmission) to seal the cuff so that no leakage occurred for 144 hours (no dye was present in the lower compartment). This was therefore set as a model operating mode where microbial proliferation was the only pathway for contaminated pooled secretions to access the lung chamber. When leakage of contaminated secretions occurred, the presence of bacteria in the lower reservoir was immediate.

### 3.2. Microaspiration Only

#### 3.2.1. Lung Fluid below the Level of the Distal Tip of the ETT

A control experiment was run at 100 cm H_2_O sealing pressure with bacterial inoculum above the cuff and sterile broth (approx. 150 mL) in the distal compartment of insufficient volume to touch the distal tip of the ETT. No growth of either MRSA or PA was detected in the lower “lung” chamber up to 144 hours (Figure 2). Additionally, results validate that the use of antimicrobial conductive paint prevented contamination of the “lung” compartment by microbial proliferation along the outer wall.

### 3.3. Microbial Proliferation Only (Cuff Overinflation)

The dye experiments verified that at cuff pressures greater than or equal to 100 cm H_2_O, microaspiration was prevented for 144 hours. The experiment with 100 cm H_2_O cuff inflation pressure, bacterial inoculum above the cuff, and fluid in the lower reservoir below the distal shaft of the ETT verified that the model design prevented bacterial access to the lower reservoir other than by proliferation along the shaft of the ETT. Figure 3 shows the results for PA containing inoculum above the cuff (inflated to a pressure of 100 cm H_2_O) and the distal tip of the ETT now submerged in a greater volume (300 mL) of sterile broth in the lung chamber (i.e., broth in the lung chamber now above the distal tip of the ETT). The “lung” chamber became positive between 24 and 48 hours of dwell. In this mode, microbial proliferation was the only mechanism by which PA could reach the “lung” chamber. Results for MRSA containing inoculum above the cuff at the high cuff inflation pressure is also presented in Figure 3. MRSA required longer than 72 hours for the “lung” chamber to become positive at the 100 cm H_2_O inflation pressure (versus greater than 24 hours for PA).

**Figure 4:**
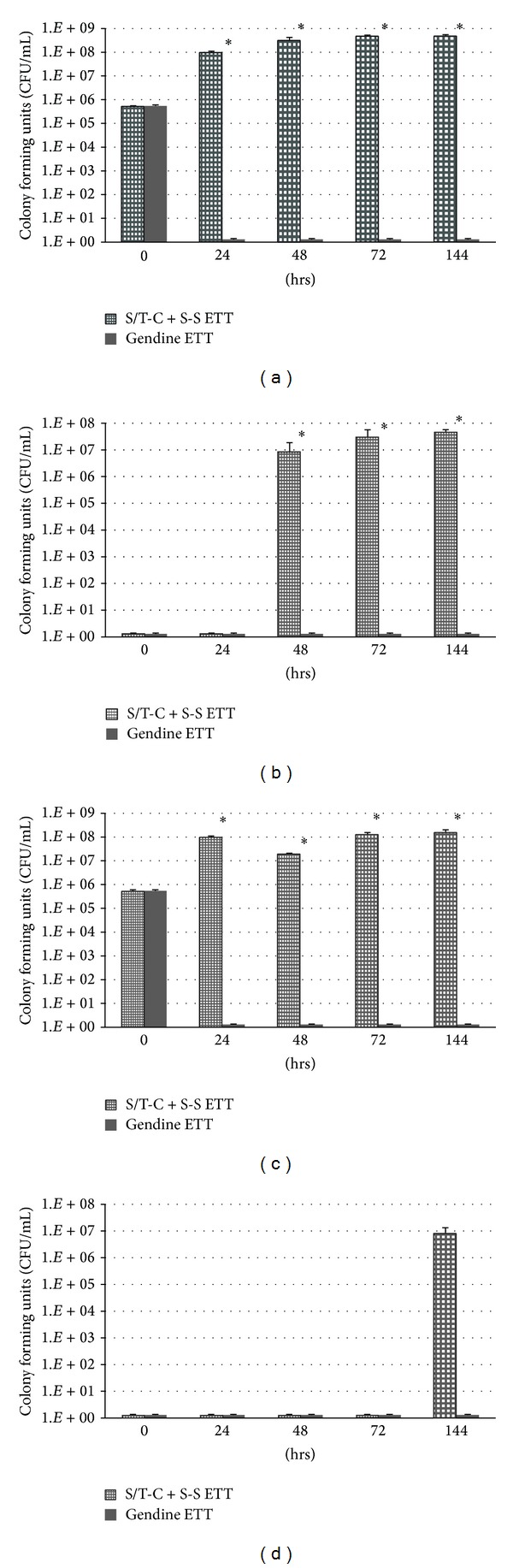
Time to “lung” contamination with gendine ETT compared to subglottic suctioning ETT. Mean and standard deviation of measured bacterial concentrations (colony-forming units [CFU]/mL) from the upper (above the cuff; (a) and (c)) and lower (“lung”; (b) and (d)) chambers in the* in vitro* model run in microbial proliferation only mode comparing quadruplicate runs of the ETT with soft-tapered cuff and subglottic suctioning (S/T-C + S-S ETT) and the antimicrobial gendine treated standard ETT (gendine ETT) at various sampling time points. The models were run with ETT cuff inflation pressures maintained at 100 cm H_2_O. Inocula of  5 × 10^5^ CFU/mL of PA (*Pseudomonas aeruginosa*) and MRSA (methicillin resistant* Staphylococcus aureus*) were pipetted on top of the cuff at time = 0 hrs. (a) above the cuff, PA; (b) below the cuff, PA; (c) above the cuff, MRSA; (d) below the cuff, MRSA. * Statistically significant pairwise differences (*P* < 0.0001).

A further set of experiments was undertaken to assess the performance of the soft, tapered-cuff ETT with subglottic suctioning (S/T-C + S-S ETT) and gendine ETT in this model. In these experiments, samples were taken from both above and below the cuff at various time points, and bacterial concentrations were quantified. Figures 4(a) and 4(b) show mean and standard deviations for four replicate runs on each ETT with PA-containing inocula added above the cuff at time 0. Figures 4(c) and 4(d) show results for analogous experiments run with MRSA.

Differences between the gendine ETT and the S/T-C + S-S ETT were found to be significant below the cuff (in the lung chamber) at 48, 72, and 144 hours for PA (*P* < 0.0001) but the study was underpowered to show statistically significant differences for MRSA (*P* = 0.06 at 144 hours). Since bacterial concentration below the cuff corresponds to the bacterial burden transmitted to the lungs, this result showed that the gendine ETT was much more effective at preventing PA and MRSA transmission by microbial proliferation. Differences in bacterial concentration above the cuff between gendine and S/T-C + S-S were also significant at 24, 48, 72, and 144 hours for both PA (*P* < 0.0001) and MRSA (*P* < 0.0001). This showed that if contaminated secretions pooled sufficiently long, the antimicrobial treatment was more effective over the testing time scales in reducing the PA and MRSA load that could potentially leak.

### 3.4. Simultaneous Microaspiration and Microbial Proliferation

**Figure 5:**
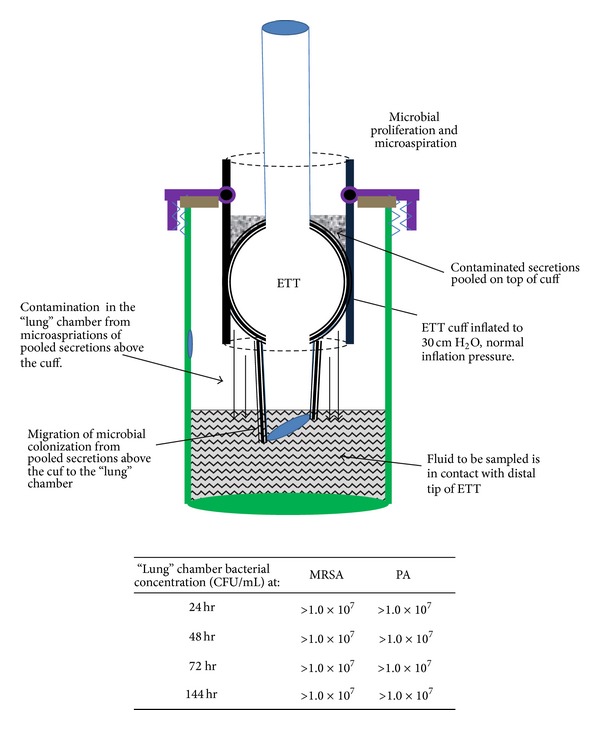
Diagram of model run in simultaneous microaspiration and microbial proliferation modes. ETT cuff inflation pressure was 30 cm H_2_O. Quantitative culture results using a standard ETT with MRSA (methicillin resistant* Staphylococcus aureus*) and PA (*Pseudomonas aeruginosa*) inocula introduced above the cuff indicate that the lung chamber became positive shortly after microaspiration occurred. Dye experiments run with the gendine standard ETT and soft tapered cuff with subglottic suctioning ETT showed that microaspiration in the model occurred within 24 hours at 30 cm H_2_O cuff inflation pressures.

For normal cuff inflation pressures (30 cm H_2_O), leakage of contaminated broth was seen within 24 hours (Figure 5). As mentioned previously, leakage was not consistently prevented unless cuff inflation pressures were maintained at 100 cm H_2_O. When leakage occurred, contamination of the initially sterile lower reservoir with bacteria was immediate and it was not possible to determine the independent contributions from each pathway.

## 4. Discussion

The focus of this model was to better understand the dual roles that aspiration and microbial proliferation can play in the pathogenesis of VAP and to determine how ETT features can contribute to interrupting those pathways. The most insight was gained by running the model in modes that isolated the effects of these mechanisms and enabled assessment of how an antimicrobial treatment as well as how (nonantimicrobial) subglottic suctioning and a tapered soft cuff could impact them. The effect of microbial proliferation was isolated by precluding microaspiration by inflating the ETT cuff to pressures in excess of physiologic which created a hydraulic seal at the cuff-tracheal junction. The effect of microaspiration was isolated by breaking the physical continuity of the proliferation pathway surface so that bacteria could not physically reach the collection fluid on the lung side of the cuff-tracheal junction. The model conditions were a worst case simulation in regard to the effects of sputum viscosity, pooled secretion volumes, and bacterial concentrations in pooled secretions above the cuff.

The microbial proliferation only mode of the model was validated by dye experiments which showed that over inflation of the cuff to 100 cm H_2_O could be sustained in a manner capable of preventing leakage through to the lungs for 144 hours. Sterility of the lung chamber in the model was validated by adding sterile broth above the cuff with over inflation and showing that the lung chamber remained sterile for 144 hours. When bacterial inocula were pooled above the cuff in this mode and when the ETT maintained a continuous physical path to the fluid in the lung chamber, as shown in Figure 3, both PA and MRSA were able to traverse the cuff-tracheal junction and contaminate the lungs. PA was able to proliferate more rapidly in this model mode and contaminated the lungs within 48 hours, while MRSA required 144 hours. Thus, at sealing pressures of 100 cm H_2_O, where microaspiration was precluded, both MRSA and PA could colonize past the cuff-trachea boundary. At normal cuff inflation pressures (which are less than 1/3 of the sealing pressures used in those experiments) neither MRSA nor PA would be prevented from colonizing to the distal end of the ETT. The mechanism by which the bacteria proliferate through a compressed boundary is unclear. The different behaviors of MRSA and PA might be due in part to the differences in their shapes, rigidities, and abilities to exert counter-pressure against surfaces.

The microaspiration only mode was validated by the experiment in Figure 2 where the lung chamber remained sterile for 144 hours when the combination of contaminated pooled secretions and cuff over inflation were unable to transmit bacteria when the physical bridge of the ETT between the two fluid chambers was broken by lowering the level of fluid in the lung chamber below the distal tip of the ETT. Because the transmission of contaminants through bulk convection is almost immediate, the effects of combined modes were dependent on the delay to where microaspiration occurred. If microaspiration occurred quickly, the lung chamber became contaminated quickly. If microaspiration took longer, then both microbial proliferation and microaspiration became contributing mechanisms.

There was rapid leakage around the cuff at 30 cm H_2_O inflation pressures for either microaspiration only mode and combined microaspiration microbial proliferation modes. In the presence of rapid leakage around the cuff, none of the ETTs prevented contamination of the lung chamber. Subglottic suctioning did affect the volume of contaminated secretions that could leak but the model was too simplified to quantify the effect of reduced volume on microaspiration. Clinically it would be expected to have a beneficial effect on reducing the bioburden in the lungs [15]. The soft cuff and tapered design had little impact on the rate of microaspiration (without subglottic suctioning) in this simplified model. Although some suggest there is sufficient evidence for these features to reduce VAP [28] others question it [29] and a recent clinical comparison did not demonstrate statistically significant reductions [30]. Little new insight was derived from the dye studies in this model into the mechanism of bacterial access to the lungs through microaspiration when tapered cuff and subglottic suctioning ETTs were employed due to model physiologic simplifications. Subglottic suctioning ETTs did not completely eliminate microaspiration because there was a layer of pooled liquid that remained below the top of the subglottic port above the cuff. Microaspiration also still occurred with tapered soft cuffs when cuff inflation pressures were not excessive. These findings are consistent with clinical studies [31]. The use of minimal cuff pressures has been advocated to minimize trauma to the trachea and impairment of blood flow in the tracheal epithelium [32]. In practice, it is difficult to ensure a hydraulic seal of these advanced cuffs to the trachea because of patient movement and development of folds and channels and due to the inability to overinflate cuffs for long periods of intubation.

When leakage was slow or precluded there were significant differences between the three ETTs. The use of the gendine antimicrobial ETT did effectively serve as a barrier to microbial proliferation in experiments where microaspiration was precluded (Figure 4). Microbially colonized ETTs can be a primary cause of VAP [32–35]. One mechanism of transmission of bacteria from the ETT to the lungs appears to be dispersion of bacterial-containing particles from the biofilm to sputum. Air in the lungs is nearly saturated with water; thus, water vapor can condense onto the luminal and external surfaces of the ETT and then contaminated condensate can drip back or be aspirated into the lungs. Since biofilms have irregular 3-dimensional structures, it is also possible for pieces of the biofilm to dislodge or shed and become inspired into the lungs during ventilation [33–35]. The gendine tube was also able to disinfect contaminated pooled secretions above the cuff in a 24-hour period (Figure 4). This is significant in that it suggests that sufficiently potent eluting antimicrobial ETTs might play a role in decreasing microbial transmission through the trachea by microaspiration if it is sufficiently delayed for the antimicrobial treatment to act on the volume of contaminated secretions pooling above the cuff. Potential irritation and toxicity from eluting gendine were previously tested* in vitro* and shown to be within acceptable limits [19] but need to be verified* in vivo*. The use of a soft cuff material, a tapered cuff design, and subglottic suctioning had little effect on proliferation of either MRSA or PA past the tracheal junction to the underside of the cuff in comparison to the standard ETT when the model was run in microbial proliferation only mode (Figure 4). Subglottic suctioning removes pooled secretions from above the suctioning port but still leaves a reasonable volume of contaminated secretions undisturbed below the suctioning port but on top of the cuff. These were probably sufficient at the bacterial concentrations in this study to promote similar rates of microbial proliferation.

New insights were obtained into potential additional benefits of an antimicrobial ETT in interfering with bacterial access to the lungs through microaspiration. Specifically the gendine ETT disinfected the pooled contaminated secretions above the cuff to the extent that when contaminated secretions pooled for sufficient duration, they converted to an essentially sterile fluid. Combining subglottic suctioning, which potentially prolongs the timescales of microaspiration with a gendine treatment, could therefore potentially further reduce bacterial access to the lungs by microaspiration substantially. Similarly, combining with advanced cuff designs and materials could further enhance the time for a gendine treated ETT to disinfect pooled secretions prior to microaspiration. The implications of these results are that ETTs that rely solely on physical and hydraulic designs to prevent bulk convection of pathogenic organisms to the lungs by microaspiration may not be sufficient to prevent all pathogenic access since microbial proliferation is an independent route. An antimicrobial ETT can play a unique role in preventing VAP in long term intubated patients by inhibiting microbial colonization as well as by disinfecting contaminated secretions which pool above the cuff.

Future work will focus on applying gendine to subglottic suctioning ETTs to better assess whether combining features can reduce transtracheal pathogen access at normal cuff inflation pressures and the minimal time required for disinfection of pooled contaminated secretions to occur and to define gendine levels that can safely contact the trachea during prolonged intubation. Future improvements to the model could incorporate flexible wall tracheas and more realistic sputum viscosity and allow for air flow and condensation of humidified air on the tube surfaces by ventilating the ETT. Future improvements will also allow more continuous monitoring and adjusting of cuff inflation pressures.
